# Turn-Taking Transitions in Conversations Among Autistic–Autistic, Non-Autistic–Non-Autistic, and Mixed-Neurotype Adult Pairs

**DOI:** 10.3390/bs16050677

**Published:** 2026-04-29

**Authors:** Zahra Poursoroush, Eugene H. Buder, Morgan Jameson

**Affiliations:** 1 School of Communication Sciences and Disorders, University of Memphis, Memphis, TN 38152, USA; zprsrush@memphis.edu (Z.P.); ehbuder@memphis.edu (E.H.B.); 2 Institute for Intelligent Systems, University of Memphis, Memphis, TN 38152, USA

**Keywords:** Double Empathy Theory, turn-taking, autism spectrum disorder, conversational timing, pragmatic language

## Abstract

**Background:** The Double Empathy Theory proposes that communication difficulties between autistic and non-autistic individuals arise from mutual misunderstandings rather than individual deficits. This study examines how autistic–autistic, non-autistic–non-autistic, and mixed-neurotype adult pairs coordinate conversations. We aimed to determine how neurotype matches or mismatches affect the types and durations of turn-taking transitions, backchannels, temporal alignment, and task performance. **Methods:** Thirty-two autistic and thirty-six non-autistic English-speaking adults were paired into autistic–autistic, non-autistic–non-autistic, or mixed-neurotype dyads. Each pair interacted virtually in a tangram task, alternating roles as describer and selector. A turn-taking coding scheme identified utterance segmentation and conversational events. **Results:** Turn-exchanges with a gap (perceived silence) were the most frequent transition type across all pairs. Matched autistic pairs produced significantly more gapless transitions than the other dyads. Mixed-neurotype dyads showed significantly longer gap durations between turns than both autistic–autistic and non-autistic–non-autistic dyads. Non-autistic–non-autistic pairs exhibited the highest proportion of backchanneling, while autistic–autistic pairs exhibited the highest proportion of simultaneous talk. Only in non-autistic–non-autistic pairs overlap frequency was associated with reduced rapport. **Conclusions:** Findings demonstrate distinct patterns in turn-taking dynamics across neurotype pairings, supporting the Double Empathy Theory highlighting the need for neurodiversity-informed rather than deficit-based approaches.

## 1. Introduction

Turn-taking is an organized system in which speakers actively manage conversational turns, requiring constant coordination among turn-takers. Turns should ideally neither start so early as to cause long overlaps, nor so late as to create awkward silences (Garrod & Pickering, 2015; Heldner & Edlund, 2010; Ruiter et al., 2006). The most efficient and coherent form of transition, reflecting close mutual monitoring during conversation between turns, is often achieved with a brief silence of approximately 200 milliseconds (Clift, 2016; Dingemanse & Liesenfeld, 2022; Heldner & Edlund, 2010; Levinson, 2016; Stivers et al., 2009).

Despite previous work on the temporal organization of turn-taking in conversation, relatively few studies have examined turn-exchange within the context of double-empathy frameworks in autism. In particular, there is a lack of research examining how specific temporal features of turn-exchange (e.g., overlaps and duration of turn-exchange) differ across different neurotype pairings, and how these patterns may reflect mutual adaptation or misalignment in interaction. The Double Empathy Theory suggests that communication difficulties between non-autistic and autistic individuals are caused, in part, by misinterpretations that arise from different communication styles, rather than a deficit on one side (Milton, 2012). Evidence supports this suggestion, showing that mixed neurotype communication partners (i.e., one autistic and one non-autistic partner) experience more communication difficulties compared to pairs of two non-autistic individuals and pairs of two autistic individuals: Mixed neurotype pairs demonstrated lower accuracy in information transfer tasks (Crompton et al., 2020; Geelhand et al., 2024; Oates et al., 2024) and lower levels of rapport during communication (Crompton et al., 2020; Foster et al., 2025; Jameson & Bean, 2025; Morrison et al., 2020) compared to autistic–autistic and non-autistic–non-autistic pairs. Neurotype-based mismatches in communication style could also influence turn-taking dynamics due to diverse interpretations of conversational signals such as timing and rhythm (De Jaegher, 2013). From a Double Empathy perspective, these differences in mutual understanding during interaction may be reflected in measurable aspects of turn-taking, including variability in the duration of turn-exchanges and the timing of overlapping speech. Such patterns may indicate differences in how individuals across neurotypes anticipate, interpret, and coordinate conversational turns.

Several studies have specifically compared turn-taking coordination in matched dyads (same neurotype) with mixed dyads (different neurotypes). Non-autistic adults adapted their speech rate to match that of their non-autistic partner during a rhythmic entrainment task; however, autistic adults did not show comparable speech-rate adaptation to non-autistic partners (Wynn et al., 2018). In a study of conversational interaction (Ochi et al., 2019), autistic adults showed longer silences (i.e., gaps) before taking their turn and a higher ratio of total pause time to speaking time during conversations with a non-autistic individual, compared to non-autistic individuals speaking with one another. During “get-to-know-you” conversations with a young non-autistic adult (Cho et al., 2023), autistic boys had longer between-turn pauses and shorter overlapping speech compared to non-autistic individuals and autistic girls. However, autistic girls showed shorter between-turn pauses and longer total durations of overlapping speech, closely resembling the transitions of non-autistic peers. Emborg (2024) found that an autistic adult took turns more frequently when his non-autistic partner allowed longer pauses, which were suggested to provide additional time for processing by the autistic individual. Rifai et al. (2022) demonstrated that both autistic–autistic pairs and mixed pairs exhibited a lower frequency of backchanneling (short affirming responses that are ≤3 syllables such as “mm-hmm”, “yeah”) compared to non-autistic–non-autistic pairs. This pattern was associated with lower rapport in mixed pairs but higher rapport in autistic–autistic pairs, illustrating another characteristic of interaction that depends strongly on the nature of the dyad, not just its participants. In contrast to studies that compared matched dyads (same neurotype) with mixed dyads (different neurotypes), a study comparing only matched dyads (same neurotype) reported that both autistic–autistic and non-autistic–non-autistic pairs preferred short-gap transitions (around 200 milliseconds (ms)) in turn-taking; autistic–autistic pairs showed significantly longer gaps in the earliest stages of conversation (mean autistic–autistic dyads = 511 ms; mean non-autistic–non-autistic dyads = 191 ms; Wehrle et al., 2023).

### Current Study

Previous research studies that have focused on the temporal coordination of turn-taking have compared only matched dyads (autistic–autistic or non-autistic–non-autistic) or mixed neurotype pairs (autistic–non-autistic) with non-autistic–non-autistic pairs. Additionally, studies that include all three pairings (autistic–autistic, non-autistic–non-autistic, and mixed neurotype pairs) have typically focused on task outcomes rather than the temporal coordination of turn-taking. Therefore, we are not aware of any research study that compares all three pair conditions of autistic–autistic, non-autistic–non-autistic, and mixed neurotype interactions in terms of temporal coordination of turn-taking, much less related aspects such as backchanneling or overlapping speech. We aim to assess and compare these conversational pairings in terms of (1) the types and durations of transitions in turn-taking; (2) backchannels and temporal alignments in conversation; and (3) relations between turn-taking dynamics and qualities of task performance including accuracy, efficiency, and rapport development.

Specific research questions are:

1. Do the frequencies of turn-exchange types (gaps and gapless turn-exchanges) differ across autistic–autistic, non-autistic–non-autistic, and mixed neurotype pairs?
2. Do the durations of gap transitions differ across autistic–autistic, non-autistic–non-autistic, and mixed neurotype pairs?
3. Do autistic–autistic, non-autistic–non-autistic, and mixed neurotype pairs differ in how often backchanneling occurs?
4. Do autistic–autistic, non-autistic–non-autistic, and mixed neurotype pairs differ in how often simultaneous talk occurs?
5. Do specific turn-taking dynamics predict the quality of task performance including accuracy, efficiency, and rapport?

We hypothesize that gapless turn-exchanges, including overlapping turn-exchanges and no pause turn-exchanges (no perceived silence), are more frequent in matched neurotypes pairs (autistic–autistic and non-autistic–non-autistic). This prediction is based on the previous literature suggesting that partners should take turns neither too soon—causing overlaps—nor too late—creating long gaps (Garrod & Pickering, 2015; Heldner & Edlund, 2010; Ruiter et al., 2006). However, we expect that mixed neurotype conversations may show longer gap transitions between turns due to misinterpretation of conversational signals by each conversation partner (De Jaegher, 2013), or because partners need more processing time to understand one another (Milton, 2012).

Rifai et al. (2022) observed that both mixed and autistic–autistic pairs used fewer backchannels; this was linked to lower rapport in mixed pairs but higher rapport in autistic–autistic pairs. Based on these findings, we hypothesize that mixed and autistic–autistic pairs will show lower frequencies of backchanneling compared to non-autistic–non-autistic pairs, suggesting that reduced backchanneling among autistic–autistic pairs could be associated with higher engagement and coordination during conversation. Simultaneous talk often reflects shared understanding and social alignment (Dunne & Ng, 1994). Therefore, we expect that mixed-neurotype pairs, due to mismatches in conversational style, will show fewer instances of simultaneous dynamics than same-neurotype pairs.

Effective performance in collaborative communication tasks relies on temporal coordination between interaction partners. Smooth turn-taking could reflect engagement and alignment between speakers at a micro-level, which may support more successful communication at a broader, task-oriented level. Conversational behaviors such as backchanneling can support shared understanding and rapport (Rifai et al., 2022). In task-oriented interactions, greater conversational alignment, including temporal and linguistic coordination, has been associated with higher task accuracy and efficiency (Geelhand et al., 2024; Jameson & Bean, 2025). Thus, we hypothesize that turn-taking dynamics associated with higher coordination, such as simultaneous talk, will be associated with better task performance in terms of accuracy, efficiency, and rapport.

## 2. Method and Materials

### 2.1. Participants

A total of 32 autistic and 36 non-autistic adults with normal hearing participated in this study. All participants used spoken English as their primary mode of communication. They were recruited based on previous study participation, word-of-mouth, academic resources such as disability service listservs, and community resources in central Ohio.

At the time of recruitment, participants completed an online informed consent form and a questionnaire including age, gender, race/ethnicity, spoken languages, education level, parental education level, and co-occurring neurodivergences. The two groups (autistic and non-autistic individuals) were broadly comparable in age distribution, with both groups concentrated in the 18–25 and 26–35 ranges and only small differences in older age ranges. They also showed overlap in educational background, with both groups spanning from high school to graduate degrees. However, some differences were observed in gender diversity and racial/ethnic representation. The inclusion of this autistic group was based on formal diagnosis as well as self-diagnosis, since there are significant structural barriers for many individuals to receive a formal diagnosis (Fletcher-Watson, 2024; Lewis, 2016; Lockwood Estrin et al., 2021; Sarrett, 2016). All participants self-reported being autistic and then answered a follow-up question indicating whether they had been formally diagnosed or self-identified. All participants also completed the Comprehensive Autistic Trait Inventory (CATI). There was no significant difference in CATI scores between formally diagnosed and self-identified autistic individuals. For more details about participants, see Table 1.

### 2.2. Recording Procedure

Each participant was paired with another individual based on overlapping availability and at least one matching demographic factor, including age group, gender, and ethnicity. Participants were paired into one of the following dyad types and were not informed of their dyad in order to prevent behavior changes based on knowing their partner’s neurotype (DeBrabander et al., 2019), and to maintain natural interaction.

1. Autistic neurotype-matched dyads: Both participants were autistic.
2. Non-autistic neurotype-matched dyads: Both participants were non-autistic.
3. Neurotype-mixed dyads: Participants had different neurotypes (one autistic, one non-autistic).

We included 20 autistic adults (10 pairs), 22 non-autistic adults (11 pairs), and 13 autistic adults paired with 13 non-autistic adults (13 pairs). The experiments were conducted virtually on the Zoom platform in one 45–60 min session. The research administrator provided participants with the link for the questionnaire and task instructions, then turned off their microphone and camera so that participants could interact and complete the task. Participants could choose whether to keep their camera on while performing the task. Five participants (two were non-autistic and three were autistic) chose not to turn on their camera, although their partners had theirs on. Despite the lack of mutual visual interaction, Pearson correlation analysis showed that the rapport between pairs was not significantly related to whether the camera was on (*p* = 0.806, r = 0.030). One participant used a mobile phone, and the rest used computers.

**Figure 1:**
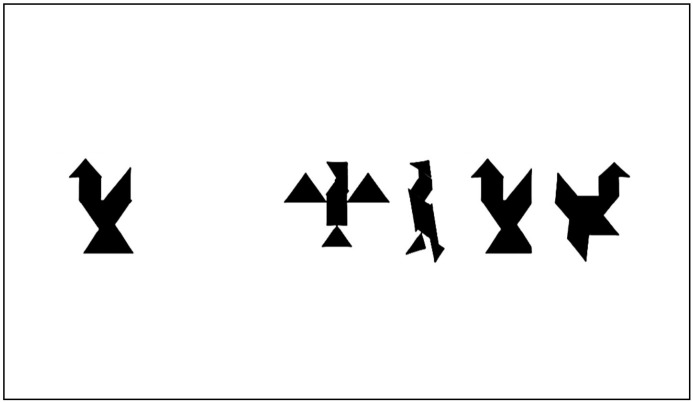
Example displays from a tangram task trial showing one target image to one participant and four response options to the other participant. Note: From Jameson and Bean (2025).

Participant pairs were asked to interact to find the target tangram together. The tangram description task was chosen because participants produced rich language, including communicating collaboratively, developing shared strategies, and creating shared understandings to perform accurately. This task also requires continuous back-and-forth coordination between partners, which naturally elicits turn-taking dynamics relevant to our measures. In each pair, one participant described a single target tangram (an abstract figure made of geometric shapes) that was shown on their screen. The other participant was the selector, who identified the tangram described by their partner among four tangram shapes (see Figure 1 for an example of the picture of tangrams in one round; Jameson & Bean, 2025). The pairs completed four rounds of four tangram sets, with each participant participating in the two roles twice (total of 16 task repetitions). Then, participants completed a self-report questionnaire about rapport. Participants rated their interaction in the questionnaire based on five aspects of rapport: ease, enjoyment, success, friendliness, and awkwardness (For more details, see Jameson & Bean, 2025).

### 2.3. Coding Procedure and Categories

Three graduate students in the field of Speech-Language Pathology coded the recordings using Action Analysis, Coding, and Training software (AACT; Delgado et al., 2010). Blinded coders used AACT to designate the appropriate category of the “Talk” and “Conversational” coding levels from Copping et al. (2023). In “Talk” coding, coders labeled intentionally communicative sounds (e.g., speech and laughter) for both participants in each pair. Following Copping et al. (2023), our criteria for determining segmentation of utterances produced by the same speaker were as follows: If the silence was less than 200 milliseconds (ms), then the two utterances were treated as a single continuous utterance. All silences greater than 300 ms were designated as gaps between talks. For silences between 200 and 300 ms, coders judged whether an utterance gap was perceptually salient or not, considering contextual factors such as surrounding speech rate.

Coding of conversational events proceeded after utterances had been located. We aimed to code dyadic interaction patterns that were built on “Talk” codes. “Conversation” code labels included:

(1) *Turn-exchange with gap* (perceived silence between one participant stopping their turn and the other starting their turn);
(2) *No pause turn-exchange* (no perceived silence between one participant stopping and the other starting);
(3) *Overlapping turn-exchange* (overlapping speech by both speakers without signs of interruption);
(4) *Overlapping turn-exchange with interruption* (one speaker could be heard to stop themselves while the other spoke);
(5) *Backchannel* (short affirming responses that are ≤3 syllables such as “mm-hmm”, “yeah” which occur during the other’s turn);
(6) *Simultaneous talk* (both speakers talk simultaneously, with one speaker’s utterance occurring within the others, such that it starts after the first speaker and ends before that speaker);
(7) *Simultaneous onset* (both speakers begin speaking at the same time based on perceptual judgment, such that one speaker ends before the other);
(8) *Simultaneous offset* (both speakers end their speech at the same moment based on perceptual judgment, such that one speaker starts later, but they finish together), and
(9) *Response* (≤3-syllable answers to questions).

**Figure 2:**
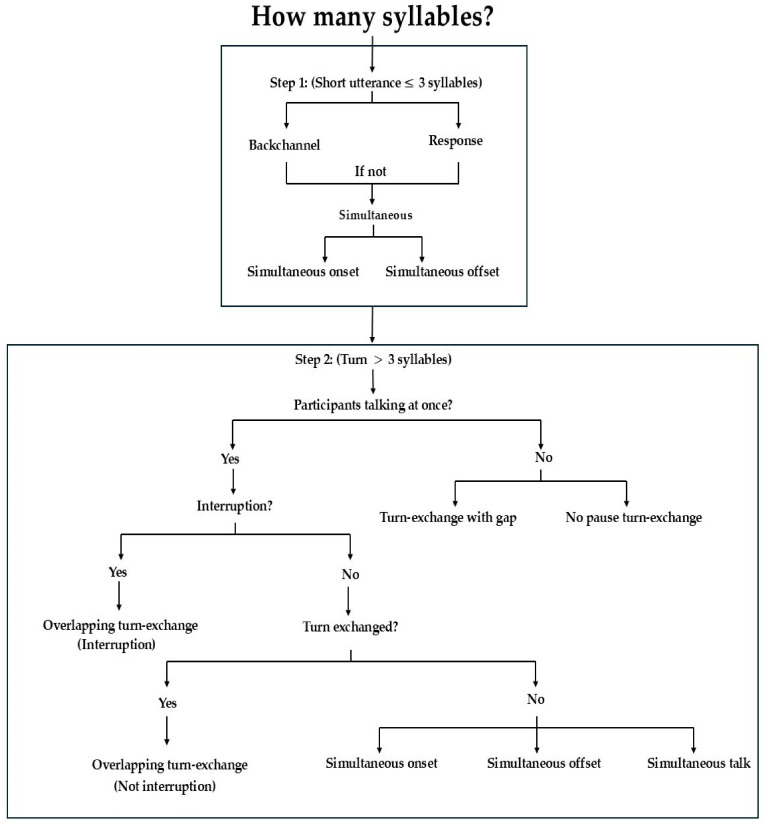
Step-by-step coding of conversational events based on utterance segmentation scaffolding.

Figure 2 shows a summary of steps for conversational events coding (for more details about the coding scheme, see Copping et al. (2023)). To assess interrater reliability, approximately 15% of the dataset was coded by two secondary coders. Segmentation of utterances employed objective durational criteria for delimiting utterances. Cohen’s Kappa between the primary coder and secondary coders ranged from 0.86 to 0.88 (Coder 1 vs. Coder 2: κ = 0.88, observed agreement = 93.4%; Coder 1 vs. Coder 3: κ = 0.86, observed agreement = 91.9%; both *p* < 0.001), indicating almost perfect agreement. While most conversational codes were defined so that each event could only be coded in one category (coders followed clear step-by-step rules to decide the correct category), they required perceptual criteria and relatively subtle distinctions, which led to some overlap between categories and affected interrater reliability. For these, Cohen’s Kappa ranged from 0.45 to 0.52 (Coder 1 vs. Coder 2: κ = 0.52, observed agreement = 60.2%; Coder 1 vs. Coder 3: κ = 0.46, observed agreement = 60.5%; both *p* < 0.001), indicating moderate agreement.

### 2.4. Data Analysis

Analyses were conducted using chi-square test of independence for research question (RQ) 1, generalized linear models (GLMs) for RQ2 and RQ5, and Kruskal–Wallis tests to evaluate group differences across autistic–autistic, non-autistic–non-autistic, and mixed-neurotype pairs for RQ3 and RQ4, using R software (Version 4.5.0; R Core Team, 2025). Before analysis, all conversational codes were exported from AACT (Delgado et al., 2010) and aggregated at the dyad level. Aggregation at the dyad level was motivated by Double Empathy Theory, which conceptualizes interactional differences as emerging from the fit between communication partners rather than from individual behavior. The total interaction time during the tangram task was approximately 6 h, including 3119 turn-exchanges across all pairs. While the neurotype condition (autistic–autistic, mixed-neurotype, and non-autistic–non-autistic) served as the consistent independent variable, the dependent variables changed according to the specific conversational metrics addressed in each RQ. For RQ1, the dependent variables were the frequencies of turn-exchange types. For RQ2, the dependent variables were gap durations treated as continuous timing measures. For RQ3 and RQ4, the dependent variable was the proportion of conversational coordination cues (backchannels, simultaneous talk, simultaneous onset, and simultaneous offset) to the total number of turn-exchanges for each pair. For RQ5, the dependent variables were task performance outcomes—accuracy, efficiency (completion time), and rapport, which were understood as warmth, mutual understanding, friendliness, and authentic feelings (Tickle-Degnen & Rosenthal, 1990).

## 3. Results

### 3.1. Frequency of Turn-Exchange Types

Across all autistic–autistic, non-autistic–non-autistic, and mixed pairs, turn-exchanges with gaps were used significantly more often than gapless turn-exchange types (including no pause turn-exchange and overlapping turn-exchange; *p* < 0.001), showing that silent transitions were the dominant form of coordination in conversation.

**Figure 3:**
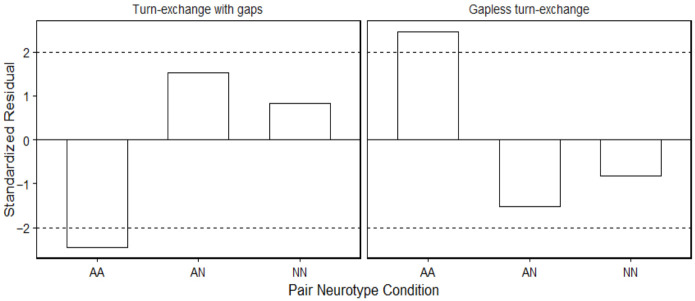
Association plot to show the relationship between pair neurotype condition (AA = autistic–autistic, AN = mixed pairs, NN = non-autistic–non-autistic pairs) and turn-exchange category. Bars above zero indicate frequencies greater than expected under independence, whereas bars below zero indicate fewer than expected. Dashed lines represent ±2, the conventional threshold for meaningful deviations.

A chi-square test of independence was then conducted to examine whether the frequency of turn-exchange types (turn-exchange with gaps vs. gapless turn-exchange) differed across autistic–autistic, mixed-neurotype, and non-autistic–non-autistic pairs. The results revealed a significant association between dyad type and turn-exchange categories (χ^2^(2) = 6.11, *p* = 0.047) although the effect size was small (Cramér’s V = 0.044), indicating that the practical significance of this association is limited despite statistical significance. Examination of adjusted standardized residuals revealed that this result was driven by autistic–autistic pairs producing significantly fewer gap transitions and more gapless transitions than expected (|*z*| = 2.46; see Figure 3) regardless of the amount of time in seconds that each pair needed to finish the tangram task (*p* = 0.923; Jameson & Bean, 2025).

### 3.2. Duration of Gap Transition

**Figure 4:**
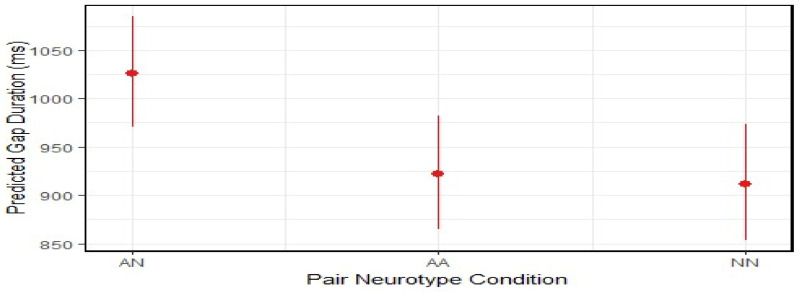
Generalized linear model results for two-way interactions (pair neurotype condition × duration of gaps transition). Error bars and shading ranges represent 95% confidence intervals (AN = mixed-neurotype pair (reference group); AA = autistic–autistic pair; NN = non-autistic–non-autistic pair).

To examine potential differences in durations of gap transitions across pair neurotype conditions, generalized linear models (GLMs) with a Gamma distribution and log link for positively skewed duration data were conducted. Model diagnostics confirmed that the assumptions of the Gamma distribution (i.e., a positive, right-skewed outcome and an appropriate mean–variance relationship) were adequately met. The analysis revealed that mixed-neurotype pairs exhibited 10–12% longer gap durations than both matched pairs (see Table 2 and Figure 4). Predicted mean gap duration was 1026 ms (95% CI [971, 1085]) for mixed-neurotype pairs, compared to 922 ms for autistic–autistic pairs (exp(β) = 0.90, 95% CI [865, 982], *p* = 0.012) and 912 ms for non-autistic–non-autistic pairs (exp(β) = 0.89, 95% CI [854, 974], *p* = 0.007).

### 3.3. Temporal Alignments in Conversation

To examine how conversational coordination and alignment differ across autistic–autistic, mixed, and non-autistic–non-autistic pairs, we calculated the proportion of conversational coordination cues (backchannels, simultaneous talk, simultaneous onset, and simultaneous offset) to the total number of turn-exchanges for each pair (the number of conversational coordination cues was divided by the total number of turn-exchanges, yielding a proportion). Expressing each cue as a proportion of total turns allowed for meaningful comparisons across pairs that differ in the number of turn-exchanges.

A Kruskal–Wallis test was conducted to determine whether the proportion of backchannels, simultaneous talk, simultaneous onsets, and simultaneous offsets differed across autistic–autistic, mixed, and non-autistic–non-autistic pairs. The results revealed significant group differences in the proportion of backchannels (χ^2^(2) = 6.58, *p* = 0.037, ε^2^ = 0.15), and simultaneous talk, (χ^2^(2) = 6.39, *p* = 0.041, ε^2^ = 0.14). However, no significant group differences were observed for simultaneous onset or simultaneous offset (*p*s > 0.35).

**Figure 5:**
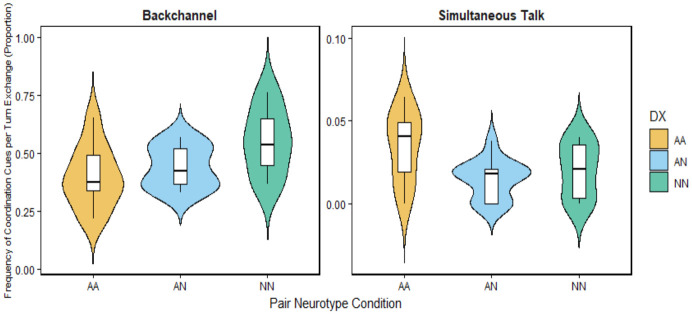
Violin plots showing the distribution of pair-level frequencies of coordination cues, expressed as proportions per turn-exchange, across pair neurotype conditions (AA = autistic–autistic, AN = mixed pairs, NN = non-autistic–non-autistic pairs).

Post hoc Dunn tests with Holm correction indicated that autistic–autistic pairs differed significantly from non-autistic–non-autistic pairs in backchannel (*p* = 0.041) and from mixed pairs in simultaneous talk (*p* = 0.036; see Figure 5). Non-autistic–non-autistic pairs exhibited the highest proportion of backchanneling (M = 0.553), followed by mixed pairs (M = 0.442), with autistic–autistic pairs showing the lowest proportion (M = 0.401). In contrast, autistic–autistic pairs exhibited the highest proportion of simultaneous talk (M = 0.035), whereas mixed pairs showed the lowest (M = 0.015), with non-autistic–non-autistic pairs intermediate (M = 0.020).

### 3.4. Relations Between Turn-Taking Dynamics and Task Performance: Accuracy, Efficiency, and Rapport

To determine whether turn-taking dynamics predicted the quality of task performance, Separate general linear models (GLMs) were conducted with accuracy, efficiency (completion time), and rapport (warmth, mutual understanding, friendliness, and authentic feelings) scores as dependent variables. Within each outcome, individual models were run for each turn-taking dynamic (e.g., laughter, overlap, response, backchannel, simultaneous onset, simultaneous offset, and simultaneous talk).

We again calculated the proportion of turn-taking dynamics (e.g., laughter, overlap, response, backchannel, simultaneous onset, simultaneous offset, and simultaneous talk) to the total number of turn-exchanges for each pair. Each model included one conversational event and its relationship with pair neurotype conditions. All model coefficients were interpreted relative to the mixed-neurotype condition, allowing comparison with matched-neurotype conditions.

All turn-taking dynamics such as laughter, overlapping turn-exchange, response, backchannel, no-pause turn-exchanges, or simultaneous speech variables were not reliable predictors of task accuracy and efficiency when we compare mixed neurotype pairs to matched neurotype pairs (autistic–autistic and non-autistic–non-autistic); both *p* > 0.05). However, low amounts of simultaneous talk were associated with substantially lower efficiency scores (needing more time to complete the task) in autistic–autistic dyads compared to the mixed dyad type (*p* = 0.023).

Rapport models revealed clearer group-related differences. Regardless of pairs’ neurotype conditions, the number of response tokens was inversely related to rapport scores: as the number of responses increased, rapport decreased (partial η^2^ = 0.21, *p* = 0.031). In addition, only non-autistic–non-autistic pairs showed significant reductions in rapport as overlapping turn-exchange increased (partial η^2^ = 0.22, *p* = 0.029), with large effect sizes compared to the mixed pairs.

## 4. Discussion

### 4.1. Summary of Findings

The present study examined how autistic–autistic, non-autistic–non-autistic, and mixed-neurotype adult pairs coordinated conversational timing during a collaborative tangram task. Turn-exchanges with gaps were the most common turn-exchange type across all pairs, confirming that brief silences are the preferred transition in all neurotypes. Because gapless turn-exchanges are associated with stronger interpersonal coordination and engagement in both autistic–autistic and non-autistic–non-autistic pairs (Garrod & Pickering, 2015; Heldner & Edlund, 2010; Ruiter et al., 2006; Wehrle et al., 2023), we predicted that gapless turn-exchanges would occur more frequently in autistic–autistic and non-autistic–non-autistic pairs. However, only autistic–autistic pairs showed a deviation from expected frequencies, producing more gapless transitions and fewer turn-exchanges with gaps. This result may suggest that autistic–autistic pairs could be more synchronized with one another, potentially due to their ability to rapidly achieve coordinated timing (Wehrle et al., 2023), or that interacting with a similar neurotype may create a more familiar or comfortable interactional contexts, which in turn may support higher coordination (Cox et al., 2025). Usage of these types of turn-exchanges by mixed-neurotype and non-autistic–non-autistic pairs did not differ from overall frequencies in this dataset.

We observed several patterns of dynamics in conversational turn-taking that reflect a significant influence of neurotype-matching or mixing. Intriguingly, mixed-neurotype pairs produced longer gap durations than both autistic–autistic and non-autistic–non-autistic pairs. This finding may suggest that participants in mixed-neurotype pairs may have required additional processing time to interpret a partner’s communicative cues that differ subtly from one’s own. The longer gap durations may indicate greater uncertainty in predicting turn boundaries, perhaps because timing or other prosodic cues were less predictable or interpreted differently across neurotypes (Levinson & Torreira, 2015). In addition, longer gap durations may also reflect speakers’ deliberate delays to avoid interrupting or overwhelming a partner. Such delays may be related to politeness or the management of social order (Brown & Levinson, 1987; Heritage, 1984) and may be more likely in mixed-neurotype interactions. Having longer gap durations in mixed-neurotype pairs is consistent with the Double Empathy Theory (Milton, 2012), which reflects a mismatch in communicative timing between speakers with different neurotypes. Longer gap durations may emerge from reduced mutual alignment in expectations about turn timing across different neurotypes.

We observed that mixed and autistic–autistic pairs used fewer backchannels compared to non-autistic–non-autistic pairs. This finding is consistent with previous research reporting that both autistic–autistic pairs and mixed pairs exhibited a lower frequency of backchanneling compared to non-autistic–non-autistic pairs, where this reduction was associated with lower rapport in mixed pairs but higher rapport in autistic–autistic pairs (Rifai et al., 2022). However, unlike the aforementioned study, we did not find any relationship between rapport and backchannel use in these pairs. The absence of a direct relationship between rapport and backchannel use in our data could support the view that rapport is not uniformly indexed by overt listener feedback across neurotypes. This could also suggest that backchanneling may be a signal of neurotype-specific social interaction, rather than solely a marker of rapport. We suggest that in autistic–autistic pairs, both participants have similar interactional styles and understand each other without the need for frequent backchanneling, resulting in lower backchannel use. In mixed pairs, backchannels are used less often because participants may be unsure of what impression they will create.

In addition, matched autistic pairs and non-autistic pairs used more simultaneous talk compared to mixed pairs. Simultaneous talk refers to moments when communication partners are talking concurrently, but neither abruptly halts as if interrupted. Such moments tend to occur when communication partners are actively participating in the interaction (Pfaender & Couper-Kuhlen, 2019). This pattern may reflect conversational cues that tend to indicate coordination, such as higher shared understanding and social alignment (Dunne & Ng, 1994) as a natural outcome of listeners accurately predicting turn endings and preparing responses in advance (Corps et al., 2018) given their mutual understanding of conversational behaviors (Milton, 2012). However, in video-mediated conversations, simultaneous talk may also reflect misaligned perceptual timing caused by latency rather than intentional coordination or interactional alignment (Seuren et al., 2021). Because our participants conversed via the Zoom platform, this may have also been a contributing factor.

When we examined how turn-taking dynamics related to communicative effectiveness, we observed that the metrics examined here were not reliable predictors of task accuracy and efficiency across neurotype pairing conditions, suggesting that variation in conversational style does not directly translate into effective communication task performances. An exception was observed for efficiency; autistic–autistic pairs were less efficient than mixed pairs when simultaneous talk was less frequent, suggesting that when both speakers talk simultaneously (without signs of interruption), this may not facilitate coordination equally across pair types. Given the results that we already found in the previous section—that autistic–autistic pairs show higher simultaneous talk, which may reflect greater shared understanding and social alignment (Dunne & Ng, 1994) in matched neurotype—we suggest that this group may require more simultaneous talk to achieve better efficiency. When they are not highly active in conversation, leading to lower efficiency, they show lower levels of simultaneous talk.

In contrast, rapport was more sensitive to conversational dynamics and pair neurotype conditions. Non-autistic–non-autistic pairs showed reduced rapport when overlapping turn-exchanges were more frequent, indicating that higher levels of overlap may be perceived as disruptive or socially misaligned within non-autistic–non-autistic interactional norms (even when most overlapping turn-exchanges are not interruptions). These findings raised the possibility that autistic and non-autistic individuals may differ in how they judge overlapping behaviors, particularly within mixed-neurotype pairs. To explore this possibility, we conducted follow-up analyses examining rapport scores related to the overlapping turn-exchange separately for autistic and non-autistic individuals within mixed-neurotype pairs involving overlapping turn-exchanges. The results revealed no significant interactions between diagnostic group (autistic vs. non-autistic) and overlapping turn-exchange (both *p* > 0.05), suggesting that neither group systematically evaluated overlap more negatively than the other within mixed interactions. This finding supports that overlapping behaviors depend on shared interactional expectations rather than a deficit on one side. Additionally, overlap in autistic–autistic and mixed neurotype pairs did not consistently signal misalignment and was not associated with rapport. Greater question response frequency (as opposed to full turns) was also associated with lower rapport across all groups, with a large effect size, suggesting that asking more short-answer questions that elicit responses of fewer than three syllables may undermine perceptions of engagement and conversational connection, regardless of neurotype.

### 4.2. Limitations and Future Studies

The findings should be used cautiously since participant demographic matching was limited in this corpus; participants were not paired on all demographic factors, including age, gender, education, employment, and race/ethnicity. In addition, the virtual platform may have shaped temporal coordination due to delays related to online communication. And finally, because this study used a cross-sectional design, it cannot capture how conversational dynamics might evolve with repeated interaction.

The coding criteria that we used to identify turn-exchanges in the present study may influence the results. Alternative criteria for turn-exchanges may yield different results, emphasizing the need for caution when generalizing these findings beyond the current coding criteria. Some of our conversational events such as no pause turn-exchange, simultaneous talk, simultaneous onset, and simultaneous offset were based on perceptual judgment rather than actual acoustic durations. This could make a difference compared to other studies that focus only on acoustic features. Future studies could also use only acoustic features and apply new analyses, such as showing the distribution of turn-exchanges with negative (e.g., overlapping) and positive values (e.g., gaps).

Future work could also extend this study using a longitudinal design, inviting the same pairs, matched on all demographic factors, to participate in multiple in-person sessions across several weeks. This could track changes in gap durations and other conversational dynamics as partners become more familiar with each other. A broader range of conversational contexts along with expanded recruitment to include a more diverse range of participants (e.g., greater gender diversity across groups), is needed to better explore communicative experiences.

### 4.3. Importance of Study

This study provides a systematic comparison of turn-exchange and conversational dynamics across autistic–autistic, non-autistic–non-autistic, and mixed-neurotype adult pairs. The findings showed that autistic–autistic pairs produced more gapless transitions and fewer turn-exchanges with gaps. Mixed-neurotype pairs demonstrated longer gap turn-exchange and reduced simultaneous talk. We also observed that rapport was sensitive to overlapping behaviors and non-autistic–non-autistic pair neurotype, highlighting that differing expectations about timing and overlap may shape social interaction. The absence of group differences (autistic vs. non-autistic individuals) in the relationship between overlapping turn-exchanges and rapport within mixed-neurotype pairs highlighted that interactional success depends on shared rules rather than deficits in one side.

These results support the Double Empathy Theory, showing that communication differences between autistic and non-autistic adults are not deficits of one group. These insights may have suggestions for education and therapy by supporting different communication styles rather than training autistic individuals to follow non-autistic norms. In addition, in the workplace, greater flexibility around conversational timing and explicit discussion of interactional expectations may promote more effective communication across neurotypes.

## Figures and Tables

**Table 1:** Demographic characteristics of participants by neurotype.

Category	Subcategory	Autistic (*n* = 32)	Non-Autistic (*n* = 36)
**Gender**	Woman	11	27
	Man	5	8
	Nonbinary	6	0
	Agender	3	0
	Genderqueer	3	0
	Gender-fluid	1	0
	Self-identify: queer, nonbinary, trans-fem	1	0
	Self-identify: trans-masc	1	0
	Not reported	1	1
**Race/Ethnicity**	Arab or Middle Eastern	1	2
	Asian	1	6
	Black or African American	0	4
	Hispanic, Latinx, or Spanish origin	1	2
	Native American or Indigenous	0	0
	White (non-Hispanic)	26	19
	Asian and White	2	2
	Not reported	1	1
**Age (years)**	18–25	19	19
	26–35	6	9
	36–45	5	3
	46–55	0	1
	56–65	0	0
	70+	0	1
	Not reported	2	3
**Education**	Did not finish high school	0	0
	High school diploma or GED	5	10
	Attended college, did not complete degree	8	5
	Associate’s degree	4	0
	Bachelor’s degree	11	9
	Master’s degree	1	8
	Doctorate or professional degree	2	3
	Not reported	1	1
**Employment**	Student	16	15
	Student + employed part-time	3	2
	Employed part-time	6	7
	Employed full-time	4	9
	Unemployed	1	1
	Self-employed	1	0
	Retired	0	1
	Other: gap year	0	1
	Not reported	0	1

Note. **Adapted from** “Implications of Linguistic Convergence and Divergence Among Matched and Mixed Autistic and Non-Autistic Communication Partners,” by M. Jameson and A. Bean, 2025, *Journal of Speech, Language, and Hearing Research*, 68(10), 4809–4828. Copyright © 2025 by the American Speech-Language-Hearing Association.

**Table 2:** Two-way generalized linear model predicting gap transition by neurotype.

Predictor	Estimate (β)	SE	*t*	*p*
Autistic–Autistic: Gap	−0.107	0.043	−2.505	0.0012
Non-autistic–Non-autistic: Gap	−0.118	0.044	−2.685	0.007

Note. Coefficients were interpreted relative to the mixed-neurotype condition (β = unstandardized coefficient; SE = standard error; *t* = t-statistic; *p* values two-tailed. Significance levels: *p* < 0.05 *, *p* < 0.01 **, *p* < 0.001 ***).

## Data Availability

The data generated and used in this study can be obtained from the corresponding author upon reasonable request.
